# Supplementary material for: Synthesis, Photophysics and Tunable Reverse Saturable Absorption of Bis-Tridentate Iridium(III) Complexes via Modification on Diimine Ligand
Source: Molecules. 2023 Jan 5;28(2):566. doi: 10.3390/molecules28020566 (PMC9864372; doi:10.3390/molecules28020566)
Supplement: Supplementary file 1 [file molecules-28-00566-s001.zip › molecules-2091065-supplementary.pdf]

Synthesis, Photophysics and Tunable Reverse Saturable Absorption of Bis-tridentate Iridium(III) Complexes *via* Modification on Diimine Ligand

Guochang Li, Zhao Jiang, Meng Tang, Xiaoli Jiang, Houfu Tu, Senqiang Zhu*, Rui Liu* and Hongjun Zhu

School of Chemistry and Molecular Engineering, Nanjing Tech University, Nanjing 211816, China

1. Experimental section

Materials

All reagents were purchased from Sinopharm Chemical Reagent Co. Ltd and used as received unless otherwise mentioned. All the high-performance liquid chromatography (HPLC)-grade solvents used for spectroscopic study were without any further purification. Ultrapure water was used in the experiments and silica column chromatography was carried out on silica gel (200-300 mesh).

Measurements

The ligands and complexes characterized by mass spectra and ^{1}H NMR. High resolution-mass (HR-MS) spectra were measured on an AB Sciex X-500B QTOF-mass spectrometer, and ^{1}H NMR spectra of the ligands and complexes in $CDCl_3$ or DMSO-d_6 solvent were measured on a Bruker Avance instrument, with tetramethylsilane for ^{1}H NMR spectra as an internal standard.

UV-vis spectra were acquired on Persee-TU-1900 UV spectrophotometer. Fluorescence spectra were recorded on a Hitachi-F4600 fluorescence spectrophotometer. Quantum yields were obtained on an Edinburgh FS-5 fluorescence spectrometer. The nanosecond time-resolved transient difference absorption spectra were detected by Edinburgh analytical instruments (LP980, Edinburgh Instruments,

(U.K.). The emission lifetimes were collected using an Edinburgh ns-lifetime instrument with a 355 nm laser as the excited source. Before each measurement, the sample solutions were degassed with N_2 for 30 min.

The nonlinear transmission experiments for Ir(III) complexes were carried out in CH_3CN solution in a 2 mm cuvette using 4.1 ns laser pulses. A Quantal Brilliant ns laser with a repetition rate of 10 Hz was used as the light source. The experimental setup and details have been described previously. An f = 10 cm planoconvex lens was used to focus the beam to the sample cuvette. The radius of beam waist at the focal point is approximately 0.101 mm, measured by a knife edge. The linear transmission of the solution was adjusted to 80%, at 532 nm in the 2 mm cuvette.

Preparation of ligands

Ligands **L1-L5** are presented in **Scheme S1**.

6-phenyl-2,2'-bipyridine (**L1**). 6-bromo-2,2'-bipyridine (234 mg, 1 mmol), phenylboronic acid (122 mg, 1 mmol), Na_2CO_3 (0.423 g, 4 mmol), toluene (30 mL) and water (10 mL) were added to a 100 mL round-bottom flask under nitrogen. The mixture was refluxed (95 °C) with stirred for 24 h, then cooled to room temperature. The residue was taken into H_2O, extracted with ethyl acetate for three times. The remnant was dried over anhydrous Na_2SO_4, and concentrated. The mixture was purified by flash chromatography on a silica gel using petroleum ether and ethyl acetate (1:2, v/v) as the eluent to give white solid (255 mg, yield 75%). ^{1}H NMR (400 MHz, $CDCl_3$) δ 8.68 (ddd, J = 4.9, 1.8, 0.9 Hz, 3H), 7.72 (td, J = 7.7, 1.8 Hz, 3H), 7.50 - 7.41 (m, 5H), 7.22 (ddd, J = 7.5, 4.8, 1.1 Hz, 5H).

2-phenyl-1,10-phenanthroline (**L2**). 2-chloro-1,10-phenanthroline (180 mg, 1 mmol), phenylboronic acid (122 mg, 1 mmol), Na_2CO_3 (0.423 g, 4 mmol), toluene (30mL) and water (10 mL) were added to a 100 mL round-bottom flask under nitrogen. The mixture was refluxed (95 °C) with stirred for 24 h, then cooled to room temperature. The residue was taken into H_2O, extracted with ethyl acetate for three times. The remnant was dried over anhydrous Na_2SO_4, and concentrated. The mixture was purified by flash chromatography on a silica gel using petroleum ether and ethyl acetate (1:2,

v/v) as the eluent to give white solid (205 mg, yield 73%). ^{1}H NMR (400 MHz, $CDCl_3$) δ 9.24 (dd, *J* = 4.3, 1.5 Hz, 1H), 8.35 (dd, *J* = 5.2, 3.3 Hz, 2H), 8.32 - 8.20 (m, 2H), 8.09 (d, *J* = 8.4 Hz, 1H), 7.78 (q, *J* = 8.8 Hz, 2H), 7.63 (dd, *J* = 8.0, 4.4 Hz, 1H), 7.55 (t, *J* = 7.5 Hz, 2H), 7.51 - 7.42 (m, 1H).

6-(9,9-dioctyl-9H-fluoren-2-yl)-2,2'-bipyridine (**L3**). 2-(9,9-dioctyl-9H-fluoren-2-yl)-4,4,5,5-tetramethyl-1,3,2-dioxaborolane (516 mg, 1 mmol), 6-bromo-2,2'-bipyridine (234mg, 1 mmol), Na_2CO_3 (0.423g, 4 mmol), toluene (30 mL) and water (10 mL) were added to a 100 mL round-bottom flask under nitrogen. The mixture was refluxed (95 °C) with stirred for 24 h, then cooled to room temperature. The residue was taken into H_2O, extracted with ethyl acetate for three times. The remnant was dried over anhydrous Na_2SO_4, and concentrated. The mixture was purified by flash chromatography on a silica gel using petroleum ether and ethyl acetate (4:1, v/v) as the eluent to give yellow liquid (535 mg, yield 70%). ^{1}H NMR (400 MHz, $CDCl_3$) δ 8.74 - 8.64 (m, 2H), 8.37 (d, *J* = 7.7 Hz, 1H), 8.20 - 8.07 (m, 2H), 7.93 - 7.83 (m, 2H), 7.81 (d, *J* = 7.8 Hz, 2H), 7.75 (d, *J* = 7.4 Hz, 1H), 7.41 (d, *J* = 7.0 Hz, 1H), 7.38 - 7.28 (m, 3H), 2.18 - 1.97 (m, 4H), 0.92 - 0.50 (m, 30H).

6-(7-([2,2'-bipyridin]-6-yl)-9,9-dioctyl-9H-fluoren-2-yl)-2-butyl-1H-benzo[de]isoquinoline-1,3(2H)-dione (**L4**). 2-butyl-6-(9,9-dioctyl-7-(4,4,5,5-tetramethyl-1,3,2-dioxaborolan-2-yl)-9H-fluoren-2-yl)-1H-benzo[de]isoquinoline-1,3(2H)-dione (692 mg, 1 mmol), 6-bromo-2,2'-bipyridine (234 mg, 1 mmol), Na_2CO_3 (0.423 g, 4 mmol) , toluene (30 mL) and water (10 mL) were added to a 100 mL round-bottom flask under nitrogen. The mixture was refluxed (95 °C) with stirred for 24 h, then cooled to room temperature. The residue was taken into H_2O, extracted with ethyl acetate for three times. The remnant was dried over anhydrous Na_2SO_4, and concentrated. The mixture was purified by flash chromatography on a silica gel using petroleum ether and ethyl acetate (4:1, v/v) as the eluent to give yellow liquid (788 mg, yield 68%). ^{1}H NMR (400 MHz, $CDCl_3$) δ 8.75 - 8.63 (m, 4H), 8.40 (d, *J* = 7.6 Hz, 1H), 8.32 (dd, *J* = 13.6, 4.7 Hz, 1H), 8.23 (d, *J* = 8.1 Hz, 1H), 8.19 (d, *J* = 5.4 Hz, 1H), 7.96 - 7.90 (m, 3H), 7.86 (dd, *J* = 13.5, 7.6 Hz, 2H), 7.75 - 7.66 (m, 2H), 7.53 (t, *J* = 4.6 Hz, 1H), 7.49 (dd, *J* = 7.7, 4.2 Hz, 1H), 7.39 - 7.33 (m, 1H), 4.32 - 4.18 (m, 2H),

2.25 - 2.03 (m, 4H), 1.82 - 1.71 (m, 2H), 1.49 (dd, J = 15.1, 7.5 Hz, 2H), 1.00 (dd, J = 15.5, 8.1 Hz, 3H), 0.91 - 0.51 (m, 30H).

9-(7-([2,2'-bipyridin]-6-yl)-9,9-dioctyl-9H-fluoren-2-yl)-3,6-di-tert-butyl-9H-carbazole (**L5**). 3,6-di-tert-butyl-9-(9,9-dioctyl-7-(4,4,5,5-tetramethyl-1,3,2-dioxaborolan-2-yl)-9H-fluoren-2-yl)-9H-carbazole (792 mg, 1 mmol), 6-bromo-2,2'-bipyridine (234 mg, 1 mmol) , Na_2CO_3 (0.423 g, 4 mmol) , toluene (30 mL) and water (10 mL) were added to a 100 mL round-bottom flask under nitrogen. The mixture was refluxed (95°C) with stirred for 24 h, then cooled to room temperature. The residue was taken into H_2O, extracted with ethyl acetate for three times. The remnant was dried over anhydrous Na_2SO_4, and concentrated. The mixture was purified by flash chromatography on a silica gel using petroleum ether and ethyl acetate (4:1, v/v) as the eluent to give white solid (771 mg, yield 65%). ^{1}H NMR (400 MHz, CDCl3) δ 9.29 (d, J = 23.5 Hz), 8.85 - 8.74 (m, 1H), 8.40 - 8.28 (m, 2H), 8.21 (d, J = 8.4 Hz, 1H), 8.17 (t, J = 2.4 Hz, 2H), 8.10 (t, J = 7.6 Hz, 1H), 7.97 (d, J = 8.0 Hz, 1H), 7.87 (d, J = 8.7 Hz, 1H), 7.83 - 7.76 (m, 1H), 7.67 (dd, J = 5.9, 4.7 Hz, 1H), 7.60 (d, J = 1.4 Hz, 1H), 7.53 (d, J = 6.4, 2H), 7.50 - 7.43 (m, 2H), 7.39 - 7.30 (m, 2H), 2.20 (d, J = 15.2, 5.0 Hz, 2H), 2.09 - 1.98 (m, 2H), 1.48 (s, 18H), 1.02 - 0.50 (m, 30H).

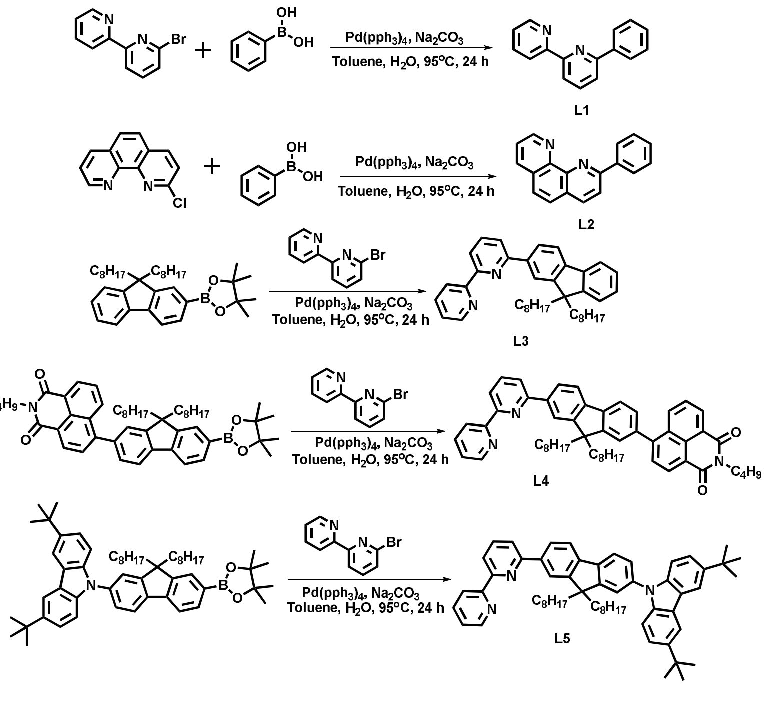

Scheme S1. The molecular structures and synthetic route to ligands **L1-L5**

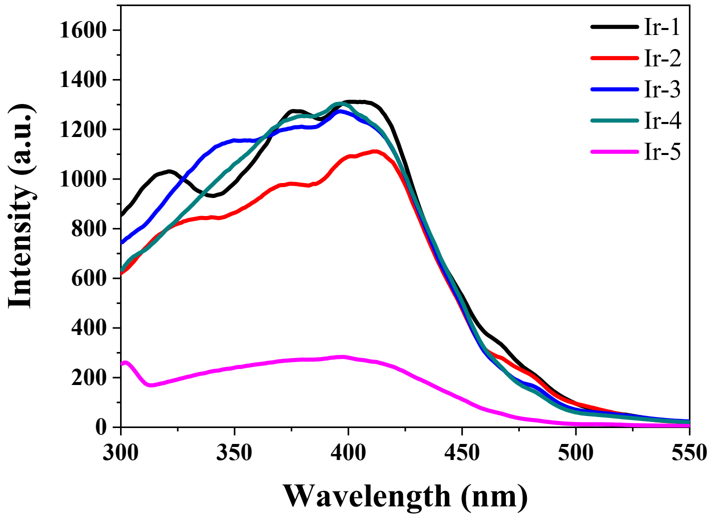

Figure S1. The excitation spectra of complexes Ir-1-Ir-5 in CH_3CN.

Table S1. Contribution of fragments in Ir complexes.

		**Contribution (%)**	
	Fragment	HOMO	LUMO
**Ir-1**	Ir	31.9	3.51
	Bipyrdine	3.69	90.4
	Benzene	3.56	0.72
	N^C^N ligand	60.8	5.37
**Ir-2**	Ir	30.8	3.54
	Phenanthroline	3.52	94.5
	Benzene	3.51	1.14
	N^C^N ligand	62.17	0.82
**Ir-3**	Ir	6.04	3.47
	Bipyrdine	9.60	94.9
	Fluorene	80.0	0.75
	N^C^N ligand	4.36	0.88
**Ir-4**	Ir	0.64	3.50
	Bipyrdine	3.01	94.81
	Fluorene	31.1	0.78
	Naphthalimide	65.0	0.01
	N^C^N ligand	0.25	0.90
**Ir-5**	Ir	0.05	3.47
	Bipyrdine	1.00	94.9
	Fluorene	12.9	0.76
	Carbazole	84.7	0.01
	N^C^N ligand	1.34	0.86

^{1}H NMR spectra and mass spectrometry

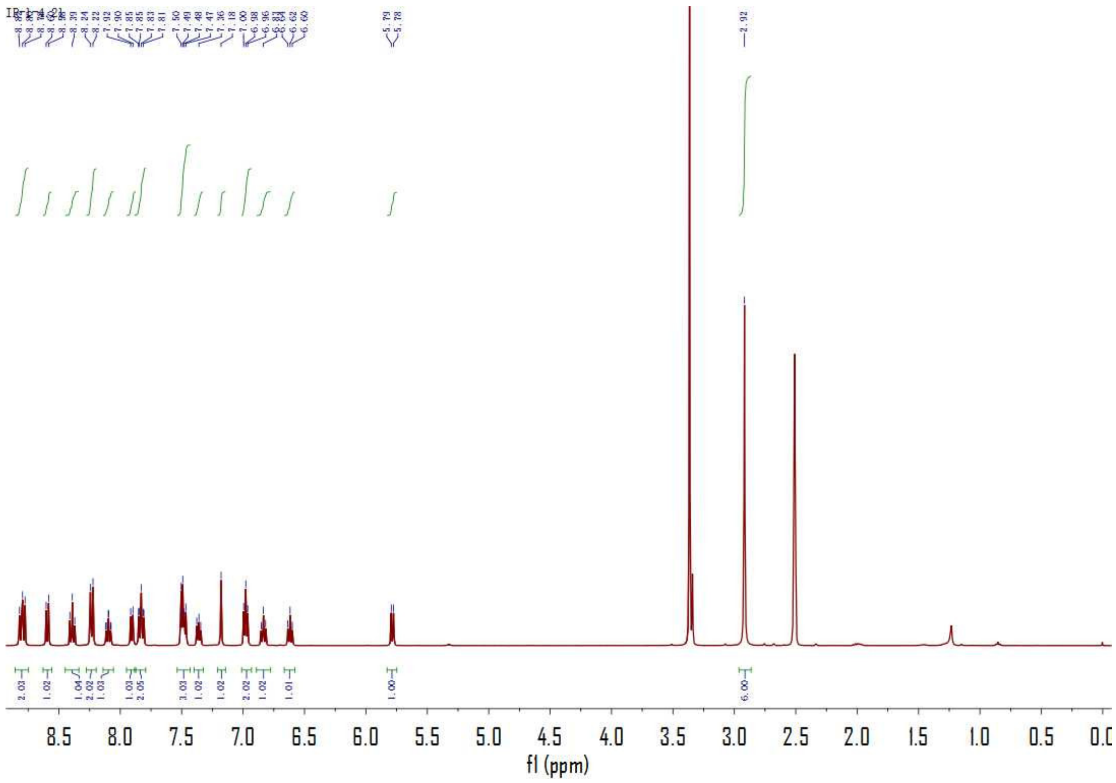

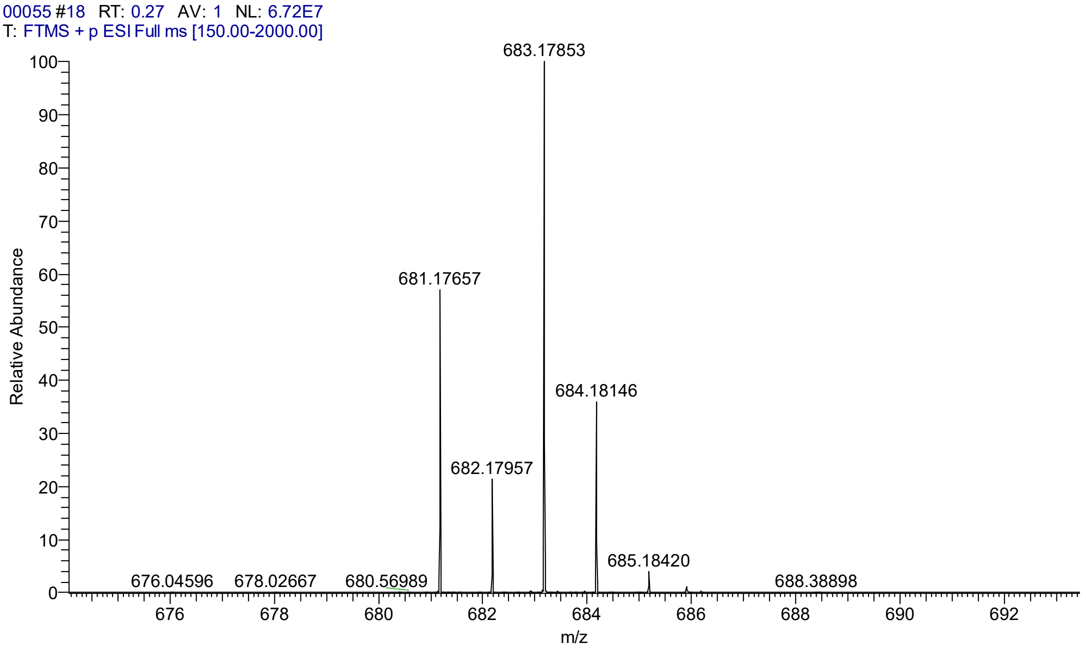

Figure. S2 ^{1}H NMR spectra (up) and mass spectrometry (down) of **Ir-1**

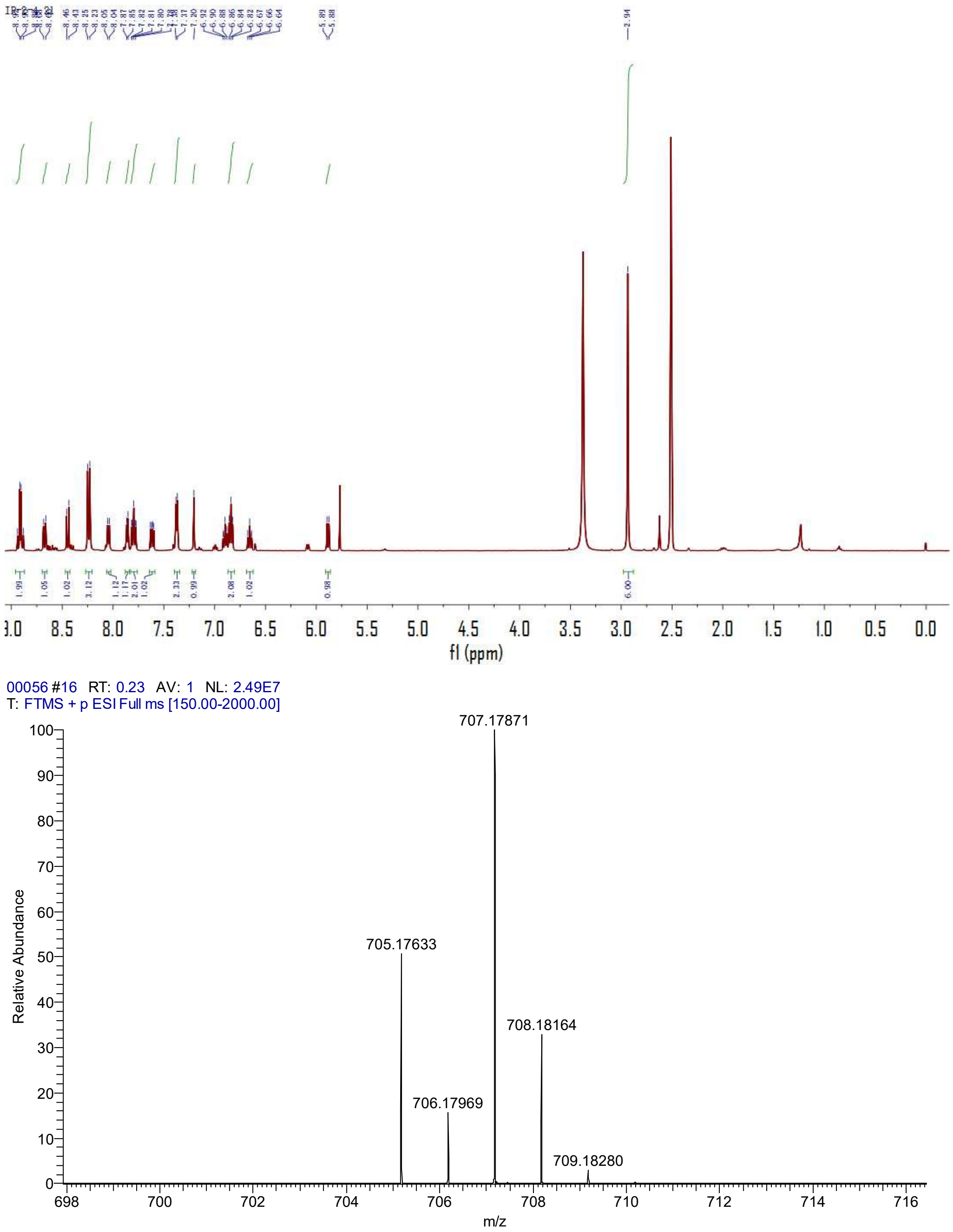

Figure. S3 ^{1}H NMR spectra (up) and mass spectrometry (down) of **Ir-2**

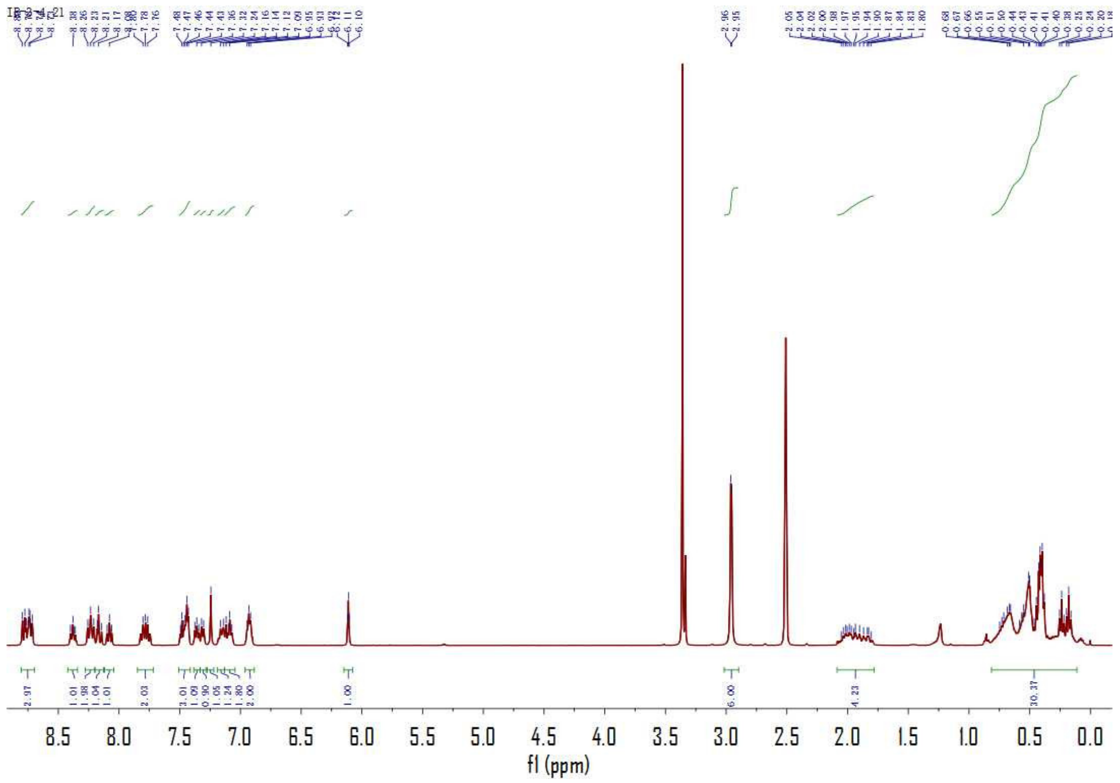

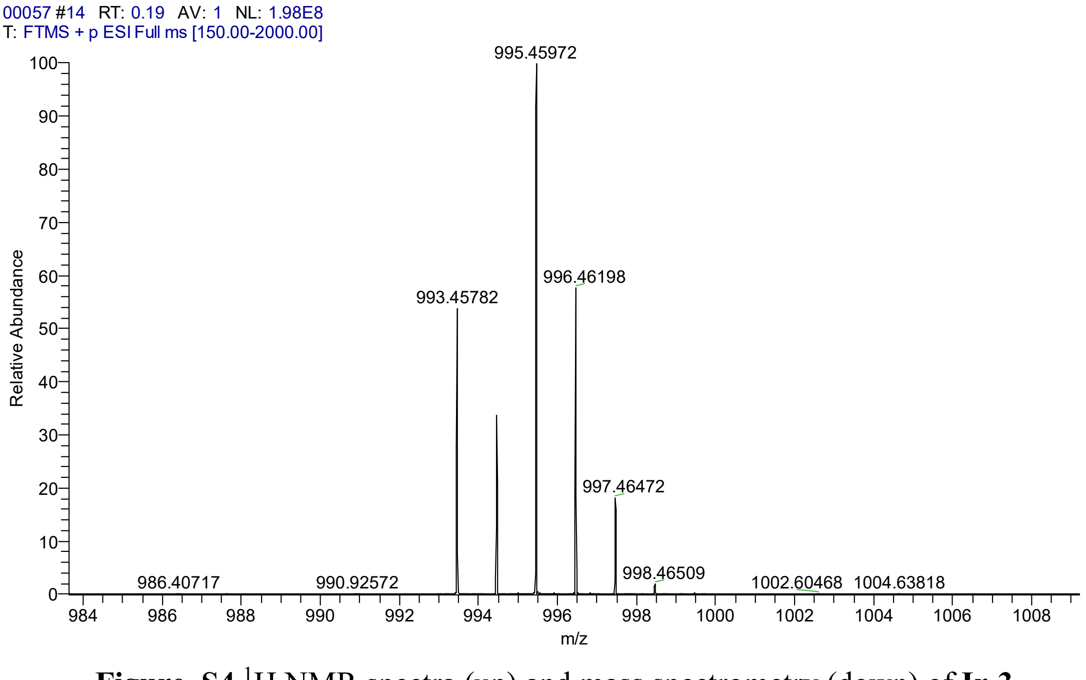

Figure. S4 ^{1}H NMR spectra (up) and mass spectrometry (down) of **Ir-3**

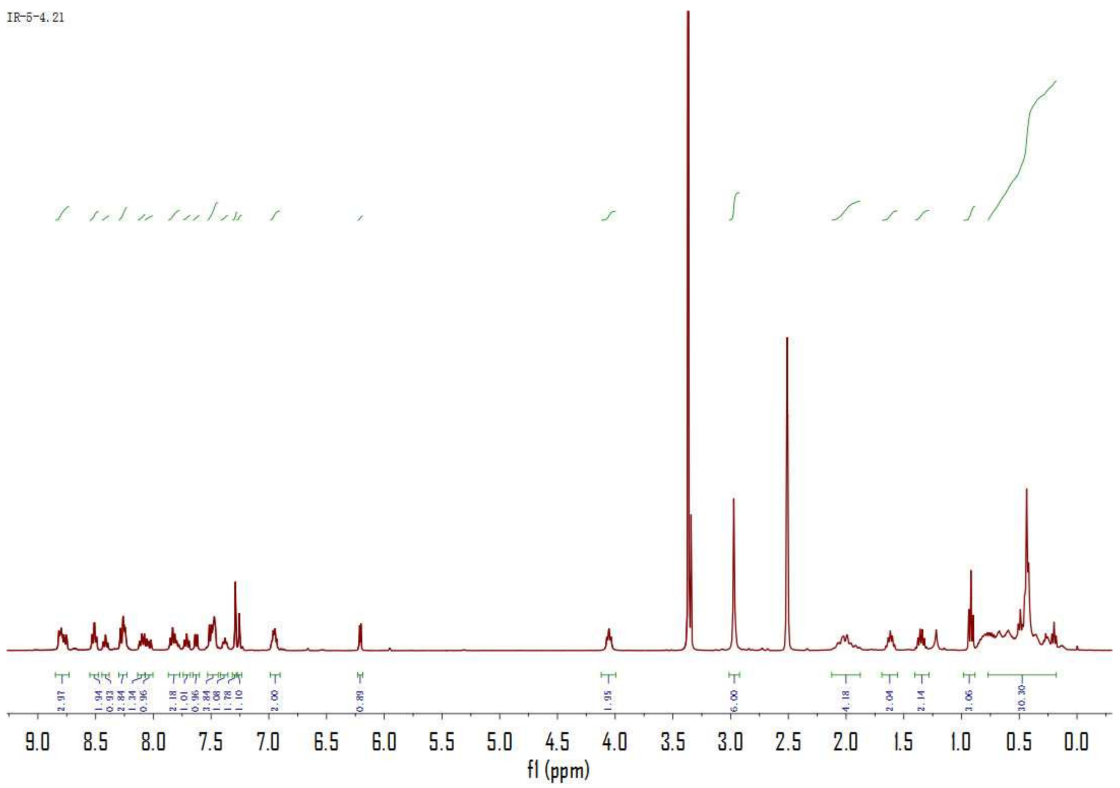

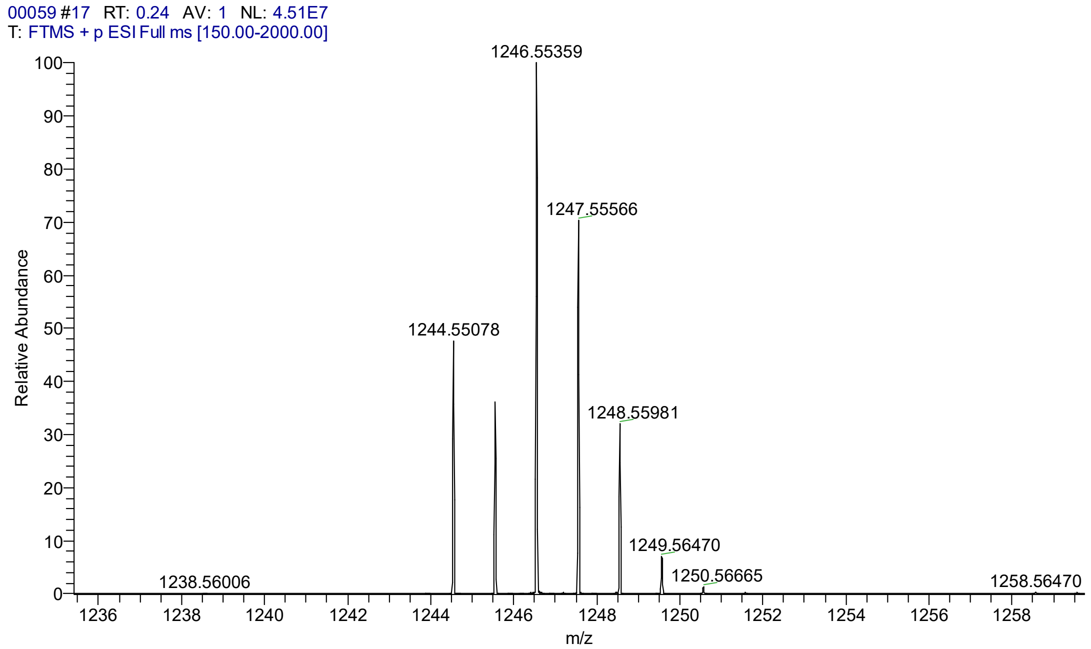

Figure. S5 ^{1}H NMR spectra (up) and mass spectrometry (down) of **Ir-4**

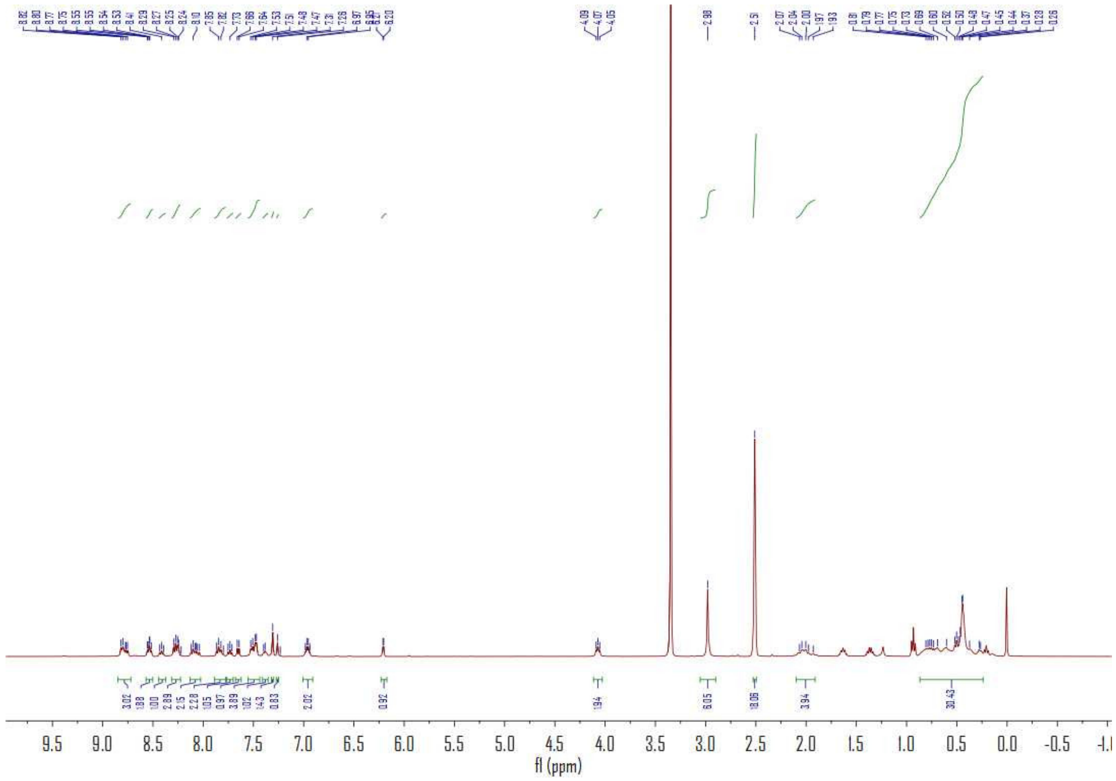

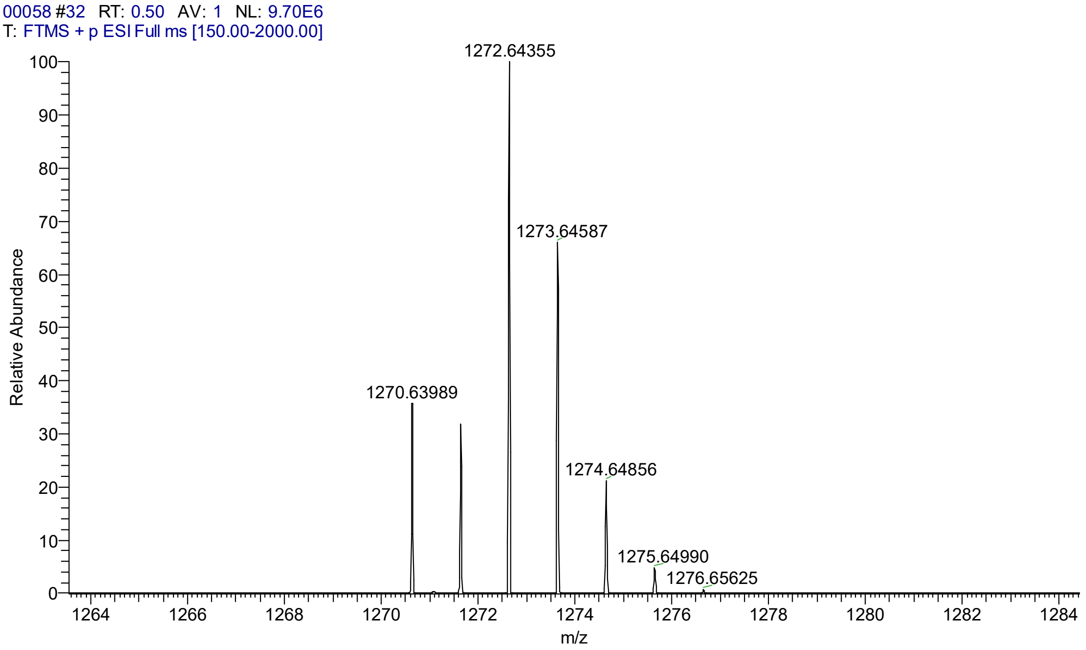

Figure. S6 ^{1}H NMR spectra (up) and mass spectrometry (down) of **Ir-5**
